# Open Anus in Certain Forms of Fæcal Incontinence

**Published:** 1844-04-06

**Authors:** 


### OPEN ANUS IN CERTAIN FORMS OF FÆCAL INCONTINENCE.

In a case of severe sporadic dysentery, recently related by M. Bouchut (Jour. de Medicine), the anus remained permanently open, and allowed the contents of the rectum to escape, without the patient being able to restrain their emission. This state of the anus he ascribes to weakness of the sphincter ani, and to predominance in the action of the levator ani, which is the dilator of the anus.

The paralysis of the sphincter ani in this case may be explained by a principle in pathology, on which great stress is laid by some continental pathologists, among whom we may mention M. Gendrin. According to this pathological law, whenever a mucous membrane is severely inflamed, the muscular fibres which are in contact with it become paralysed, as a necessary consequence of the propagation to them of the inflammation. Thus, when the mucous membrane of the larynx is inflamed, the muscular fibres of the vocal cords are paralysed, and aphony follows. The existence of the law may be illustrated by many other examples. In bronchitis, or inflammation of the bronchial mucous membrane, if the inflammation is intense, extreme dyspnœa follows, and as M. Gendrin very correctly remarks, all the symptoms of pulmonary emphysema ensue, owing, no doubt, to the paralysis of the muscular fibres of the bronchial tubes. In peritonitis the inflammation of the peritoneal covering of the intestinal canal extends to the muscular coat, completely paralyses it, and thus gives rise to constipation; as, also, the paralysed intestine no longer offers any resistance to the expansion of the gases it contains, tympanitis follows. In severe enteritis it is the inflammation of the mucous membrane which paralyses the muscular structure; but as the intestinal mucous membrane is separated from the muscular one by a fibro-cellular layer, the inflammation acts less intensely on the muscular fibres than in peritonitis, and unless it be very severe, diminishes, but does not abolish, its contractile power; thence it is that generally speaking in enteritis we have mere constipation, tympanitis only existing in the worst cases.—*Ibid.*

